# Consequences of Interdialytic Weight Gain Among Hemodialysis Patients

**DOI:** 10.7759/cureus.15013

**Published:** 2021-05-13

**Authors:** Mojgan Jalalzadeh, Seyednouraddin Mousavinasab, Camila Villavicencio, Muhammad Aameish, Shobhana Chaudhari, Donald Baumstein

**Affiliations:** 1 Internal Medicine/Nephrology, Metropolitan Hospital Center, New York Medical College, New York, USA; 2 Advanced Statistical Modeling, Mazandaran University of Medical Sciences, Mazandaran, IRN; 3 Internal Medicine, Metropolitan Hospital Center, New York Medical College, New York, USA; 4 Internal Medicine/Geriatrics, Metropolitan Hospital Center, New York Medical College, New York, USA

**Keywords:** interdialytic weight gain, hemodialysis, high blood pressure, nutritional status

## Abstract

Background

Interdialytic weight gain (IDWG) is a marker of higher pre-dialysis blood pressure, nutrition, and survival in hemodialysis (HD) patients. However, this relationship is incompletely characterized. In this study, we seek to define the association of IDWG/dry weight x100 (IDWG%) on blood pressure (BP), and the nutritional status of an HD population.

Material and Methods

This study was performed on 300 HD patients. The data was collected over four weeks, including total IDWG, IDWG%, and blood pressure. Normalized protein nitrogen appearance (nPNA), and serum albumin were used as markers of nutritional status. Participants were divided into three groups according to the mean of the IDWG% between two sessions of HD (group A < 3%, group B = 3% - 3.9%, and group C ≥ 4%); they were then compared on various aspects. Student t-test, analysis of variance (ANOVA), and linear regression analysis were used as statistical tools.

Results

The mean (± standard deviation (SD)) age was 61.7 ± 14.2 years with 57.7% of the patients being male and 42.3% being female. The mean IDWG% for the whole studied population was 3.72% ± 1.73%. Between these three groups, a higher IDWG% was associated with younger males (p = 0.032), lower dry weight (p = 0.009), and longer duration on HD therapy (p = 0.009). IDWG% was directly associated with lower pre-dialysis serum sodium (p = 0.04), higher pre-dialysis serum creatinine (P = 0.002), and lower body mass index (BMI) (p= 0.003). Between these three groups, interdialytic variations in weight gain were not associated with increased BP. There was no significant difference between the three groups in terms of nPNA and serum albumin.

Conclusions

The most important associations of IDWG% are age, weight, pre-dialysis sodium, serum creatinine, and duration of dialysis (months). There was no association between IDWG% and increased systolic BP. IDWG% had no association with nutritional status.

## Introduction

At all stages of chronic kidney disease (CKD), sodium and water overload may cause plasma volume expansion, left ventricular (LV) dilatation, and LV hypertrophy. This is particularly seen in dialysis patients [1-2]. Hypertension (HTN) and malnutrition are common problems among patients undergoing chronic hemodialysis (HD). HTN is often uncontrollable in HD patients. Causes of HTN are multifactorial, but there is a direct relationship between volume and HTN.

Interdialytic weight gain (IDWG) is used as a parameter for fluid and salt intake between the two sessions of HD. IDWG was measured as the pre-dialysis weight prior to HD treatment, minus the prior session post-dialysis weight. It influences blood pressure and survival of HD patients [3-7]. However, the extent to which interdialytic volume gains affect BP in HD patients is less clear and remains controversial [3, 8-9]. Fluid restriction, longer sessions of dialysis, frequent dialysis sessions, and correction of sodium dialysate concentrations have been suggested to reduce IDWG [10]. Dietary salt restriction is necessary for the dialysis population in the management of HTN and IDWG [11].

At the same time, IDWG is a marker of nutrition in HD patients [3]. Malnutrition among HD patients can lead to an increased morbidity and mortality rate [12-13]. Daily protein intake, estimated by normalized protein nitrogen appearance (nPNA), is calculated from urinary nitrogen output and non-urea nitrogen losses. Dialysis should be started when the nPNA falls below 0.8 g/kg per day to prevent deterioration of nutritional status and leads to a better clinical outcome. Also, when nPNA in HD maintenance patients is low, it usually indicates a low dietary intake [14]. Reasons for malnutrition in dialysis patients include decreased appetite and food intake, chronic inflammation, and decreased physical activity [15]. Several studies have shown that higher IDWG is associated with improvement of nutritional status [3, 5-6].

Herein, we sought to identify the influence of IDWG% and not IDWG, as done in many other studies on BP and nutritional status in HD patients. IDWG% is IDWG/dry weight x100, and dry weight (DW) is the patient's weight without the excess fluid that builds up between dialysis treatments.

## Materials and methods

This prospective study was conducted on 300 HD patients at four HD centers in the Provinces of Zanjan and Tehran in Iran for one month. The study was approved by the Research Ethics Committee of the Zanjan University of Medical Science (approval #08/90-327-02). All patients signed a consent form prior to data collection.

The association of IDWG% (as a percentage of IDWG/dry body weight) on BP and nutritional status was investigated. Inclusion criteria included patients aged ≥ 16 years with more than six months on maintenance HD three times weekly. Incomplete data at analysis time were considered as exclusion criteria. Participants were divided into three groups according to IDWG%: Group A < 3%, Group B = 3% - 3.9 %, and Group C > 4% in between two sessions of dialysis. All examination and laboratory values were collected when patients reached their dry weight. Dry weight was defined to achieve an edema-free state and no orthostatic hypotension at the end of the HD session. IDWG was calculated as the pre-dialysis weight prior to HD treatment, minus the prior session post-dialysis weight. Blood pressure was measured manually by the staff of the dialysis unit shortly before the HD session, and the weight was measured before and after HD treatment. IDWG and blood pressure were averaged for pre-dialysis measurement of three hemodialysis sessions per week for four weeks and were used for statistical analysis. The serum albumin level and nPNA, which reflect the daily protein intake, were used to evaluate nutritional status. The nPNA was calculated with the equation of Bergström, nPNA = 0.45 Kt/V_urea_ + 0.38 (urea clearance normalized to total body water). Individuals who prescribed antihypertensive medications and with blood pressure more than 140/85 were considered to have hypertension. Serum pre-dialysis cholesterol, triglycerides, fasting glucose, hemoglobin (Hgb), albumin (Alb), calcium, phosphorus, and C-reactive protein (CRP) were measured. To evaluate the efficacy of dialysis, Kt/V was calculated. Dialysis duration (defined as a month of dialysis therapy), age, and gender were extracted from patients’ records and saved. The HD protocol for all patients was four hours of HD using hemophane membranes, with a blood flow rate of 300 - 350 mL/min and dialysate flow rate of 500 - 700 mL/min, bicarbonate bath, on a thrice-weekly schedule. All our patients were dialyzed with a sodium dialysis concentration of 139 mmol / L.

Statistical analysis 

Data were reported as mean ± standard deviation (SD) for continuous variables with normal distributions and numbers (percent) for categorical data. Demographic characteristics, laboratory data, and blood pressures were categorized into three groups of IDWG%. Differences between these groups were analyzed with analysis of variance (ANOVA) followed by Tukey’s honestly significant difference (HSD) post-hoc test or the Kruskal Wallis nonparametric test as appropriate. For categorical data, the Pearson Chi-squared test was used. IDWG or IDWG% was entered as a response variable. Spearman correlation and multivariate linear regression analysis were performed to identify associations between IDWG or IDWG% with systolic and diastolic BP and other variables, including various nutritional parameters. The following possible explanatory variables were entered into the model: age, gender, weight, height, Kt/V, serum albumin, and pre-dialysis plasma sodium concentration. Also, bivariate analyses were used to independently identify variables associated with systolic BP. Then, significant factors in bivariate analysis with a P-value less than 0.5 were entered into a multivariable model to describe the relationship between BP with IDWG% and each clinical and demographic characteristic. Statistical analyzes were performed with the IBM Statistical Package for Social Sciences (SPSS), version 20 (IBM SPSS Statistics for Windows, Armonk, NY). Two-tailed P-values < 0.05 were considered statistically significant.

## Results

Three hundred HD patients were enrolled in this study, including 173 males (58%) and 127 females (42.3%) with a mean age of 61.77 ± 14.2. High blood pressure was detected in 83.7% (251 patients) and diabetes mellitus (DM) in 52% (156 patients). The mean BMI, weight gain, and IDWG% were 24.07± 4.13, 2.32 kg ± 1.1, and 3.72 %± 1.73, respectively. Table 1 shows the demographic data of the patients.

**Table 1:** Demographic, Metabolic, and Laboratory Features of Patients on Hemodialysis IDWG and blood pressure were averaged for pre-dialysis measurement of three hemodialysis sessions per week for four weeks. BMI: body mass index; BP: blood pressure; CRP: C-reactive protein; HDL: high-density lipoprotein; IDWG: current predialysis weight (kg) – previous postdialysis weight (kg); IDWG%: current predialysis weight (kg) – previous postdialysis weight (kg)/target dry weight (kg) x100; Kt/V: urea clearance normalized to total body water; nPNA: normalized protein nitrogen appearance; SD: standard deviation

Variable	Value
Number	300
Sex (Male/Female%)	173 (58%), 127 (42.3%)
Age (mean ± SD) years	
Male	61.27 ± 14.7
Female	62.45 ± 13.5
Height (cm)	166.1 ± 8.7
Diabetes mellitus	156 (52%)
Cardiovascular events	63(21%)
Cerebrovascular accident	72(24%)
Metabolic features	
Hypertension	251 (83.7%)
Systolic BP (mm Hg)	140.9 ± 18.2
Diastolic BP (mm Hg)	83.6 ± 9.1
Low HDL	145 (48.3%)
Abnormal glucose metabolism	140 (46.7%)
Elevated triglycerides	103 (34.3%)
Abdominal obesity	124 (41.3%)
Measurements	
Dry weight (kg), mean ± SD	63.8 ± 12.5
Interdialytic weight gain (IDWG) (kg), mean ± SD	2.32 ± 1.1
IDWG% (IDWG/DW, %), mean	3.72 ± 1.73
Serum laboratory features	
Fasting blood sugar (mg/dL)	121.4 ± 61.7
Cholesterol (mg/dL)	157.7 ± 42.1
Triglyceride (mg/dL)	149.7 ± 90.5
HDL, mg/dL	44.8 ± 10.6
Blood urea nitrogen (mg/dL)	76.7 ± 46.5
Creatinine (mg/dL)	8.7 ± 2.5
Sodium (mEq/L)	138.8 ± 3.5
Calcium (mEq/L)	9.03 ± 0.7
Phosphorus (mEq/L)	4.9 ± 1.2
Albumin (mg/dL)	4.0 ± 0.56
CRP	20 (0 - 120)
Duration of dialysis (months), median (min-max)	48 (12 - 192)
Kt/V	1.21 ± 0.25
nPNA (g/kg/day)	0.92 ± 0.1
BMI	24.07 ± 4.1

Table 2 shows the clinical results and the analysis of the three groups of the patients according to the IDWG%. Thirty-six percent were in group A (IDWG% < 3%), 24% in group B (IDWG%: 3% - 3.9%), and 40% in group C (IDWG% ≥ 4%). Higher systolic pre-dialysis blood pressure was detected in 78.5% of patients in group A, in 89% of patients in group-B, and in 85.0% of patients in group-C. There was no difference between three groups in terms of BP (P = 0.15), nutritional elements of nPNA, and serum albumin (p = 0.41). IDWG% was significantly higher in younger males (p = 0.027), those with lower DW (p = 0.009), lower BMI (p = 0.003) and longer duration on HD therapy (p = 0.009). IDWG% was not associated with height (P = 0.95). Patients with higher IDWG% had lower level of pre-dialysis serum Na (p = 0.04), and higher pre-dialysis serum creatinine (p = 0.002).

**Table 2:** Clinical and Analytical Characteristics of 300 Patients According to the Three Groups of Percentage Interdialytic Weight Gain/Dry Weight (IDWG%) Results are expressed as mean ± standard deviation (SD) or median (min-max). Statistical comparison was performed with analysis of variance (ANOVA), Kruskal Wallis, or Chi-squared tests. IDWG = current predialysis weight (kg) – previous postdialysis weight (kg); IDWG% = current predialysis weight (kg) – previous postdialysis weight (kg)/target dry weight (kg) x100 BMI: body mass index; BP: blood pressure; CRP: C-reactive protein; Kt/V: urea clearance normalized to total body water; nPNA: normalized protein nitrogen appearance; SD: standard deviation

Variables		Group A IDWG% < 3	Group B IDWG% (3 - 3.9)	Group C IDWG% ≥ 4		P-value	
Number of patients, %	107 (35.7%)		73 (24.3%)	120 (40%)	-		
Gender (Female/Male)	44/63		31/42	52/68	0.94		
Age (mean ± SD)	63.27 ± 14.8		63.16 ± 13.8	59.58 ± 13.7	0.039		
Male’s age	64.13 ± 13.8		62.29 ± 15.3	58.00 ± 14.7	0.027		
Female’s age	62.05 ± 16.8		64.35 ± 11.5	61.65 ± 12.2	0.72		
Height	164.2 ± 8.9		164.11 ± 7.7	163.9 ± 9.0	0.95		
Diabetes mellitus	58 (54.2%)		43 (58.9%)	55 (45.8%)	0.18		
Hypertension	84 (78.5%)		65 (89.0%)	102 (85.0%)	0.15		
Measurements							
Dry weight, mean ± SD	65.6 ± 13.1		65.5 ± 11.4	61.1 ± 12.2	0.009		
Interdialytic weight gain (IDWG) (kg), mean ± SD	1.34 ± 0.6		2.28 ± 0.4	3.25 ± 0.8	< 0.001		
IDWG%, mean	2.01 ± 0.8		3.48 ± 0.3	5.38 ± 1.2	< 0.001		
Outcome							
Pre-dialysis systolic BP, mm Hg	130 (80 - 200)		130 (90 - 200)	130 (90 - 180)	0.38		
Pre-dialysis diastolic BP, mm Hg	80 (50 - 110)		80 (50 - 100)	80 (50 - 110)	0.81		
nPNA (g/kg/day)	0.91 ± 0.1		0.93 ± 0.08	0.94 ± 0.12	0.11		
Serum laboratory features							
Blood urea nitrogen (mg/dL)	63 (17 - 242)		67 (19 - 243)	62 (19 - 259)	0.43		
Creatinine (mg/dL)	8.01 ± 2.4		8.86 ± 2.7	9.17 ± 2.5	0.002		
Sodium (mEq/L)	139.5 ± 3.5		138.4 ± 3.3	138.5 ± 3.5	0.04		
Calcium	8.9 ± 0.7		9.15 ± 0.6	9.07 ± 0.7	0.085		
Phosphorous	4.8 ± 1.1		5.02 ± 1.3	4.9 ± 1.2	0.63		
Albumin (mg/dL)	4.0 ± 0.7		4.0 ± 0.5	4.0 ± 0.5	0.96		
CRP	0 (0 - 120)		20 (0 - 120)	20 (0 - 80)	0.97		
Duration of dialysis (months), median (min-max)	36 (12 - 120)		36 (12 - 144)	48 (12 - 192)	0.009		
Kt/V	1.18 ± 0.25		1.22 ± 0.18	1.25 ± 0.27	0.11		
BMI	24.3 ± 4.3		24.3 ± 3.9	22.6 ± 3.8	0.003		

Table 3 shows the correlation between IDWG% and numerical measurements. There was a significant inverse correlation between IDWG% with age (r = −0.129, P = 0.026), dry weight (r = −0.173, P = 0.003), waist circumference (r = −0.128, p = 0.026), BMI (r = −0.180, p = 0.002), pre-dialysis serum sodium (r = −0.151, P = 0.009), and a positive correlation with the duration of dialysis therapy (r = 0.164, P = 0.004). There was no significant correlation with systolic and diastolic BP. All of the results in this analysis are consistent with the results in Table 2.

**Table 3:** The Association of Clinical and Analytical Characteristics with IDWG% *Spearman correlation BMI: body mass index; BP: blood pressure; IDWG%: interdialytic weight gain/dry weight x100

IDWG%		
Variables	r*	P
Age	-0.129	0.026
Waist circumference	-0.128	0.026
BMI	-0.180	0.002
Duration of dialysis	0.164	0.004
Creatinine	0.200	< 0.001
Sodium	-0.151	0.009
Alkaline phosphatase	0.198	0.001
Dry weight	-0.173	0.003
Systolic BP	-0.020	0.730
Diastolic BP	-0.035	0.544

Table 4 shows the correlation between systolic and diastolic BP and numerical measurements. Young age was correlated with higher BP (r = -0. 177, P = 0.002) in the univariate analysis. However, sex, diabetes, and longer duration of dialysis were not associated with high BP. There was a significant inverse correlation between systolic BP with hemoglobin (Hgb) (r = −0.202, P < 0.001) and hematocrit (Hct) (r = −0.169, p = 0.003), as well as a positive correlation with fasting blood sugar (FBS) (r = 0.152, p = 0.008) and blood urea nitrogen (BUN) (r = 0.127, p = 0.028). The same correlations also were found for diastolic BP.

**Table 4:** The Association of Clinical and Analytical Characteristics with Systolic BP and Diastolic BP *Spearman correlation BP: blood pressure; BUN: blood urea nitrogen; FBS: fasting blood sugar

	Systolic BP		Diastolic BP	
Variables	r*	P	r*	P
Age	-0.177	0.002	-0.153	0.008
FBS	0.152	0.008	0.132	0.022
BUN	0.127	0.028	0.179	0.002
Hemoglobin	-0.202	< 0.001	-0.158	0.006
Hematocrit	-0.169	0.003	-0.129	0.026

Table 5 shows the variables that have a statistically significant effect on systolic BP by a stepwise regression method. In bivariate analysis, factors with a P-value < 0.5 were entered into a multivariable model. Since the P-value of the IDWG% was not less than 0.5, it was not entered into a multivariable model. Increased hemoglobin and hematocrit level and age were associated with lower pre-dialysis systolic BP. In multivariable analyses, greater hemoglobin remained a significant predictor of lower pre-dialysis systolic BP with a coefficient of r^2^ = 0.03.

**Table 5:** Bivariate and Multivariable Predictors of Pre-dialysis Systolic Blood Pressure in Hemodialysis Patients Note: Values expressed as mean ± standard error (SE). Selected multivariable regression models were done by a stepwise method. BUN: blood urea nitrogen; Ca: calcium; CRP: C-reactive protein; DM: diabetes mellitus

Variables	Bivariate Parameter Estimates of Slope	P-Value	Multivariable Parameter Estimate of Slope	P-Value
Hemoglobin	-1.77 ± 0.55	0.001	-1.76 ± 0.55	0.001
Age	-0.157 ± 0.07	0.034		
Hematocrit (%)	-0.44 ± 0.17	0.013		
BUN (mg/dL)	0.04 ± 0.02	0.075		
Ca (mg/dL)	2.41 ± 1.48	0.105		
Triglycerides (mg/dL)	-0.018 ± 0.01	0.114		
Potassium (mEq/L)	1.99 ± 1.4	0.148		
Abnormality glucose (> 126 vs < 126)	2.79 ± 2.1	0.183		
Ferritin (ng/mL)	-0.002 ± 0.002	0.290		
Height (cm)	0.122± 0.12	0.312		
Phosphorous (mg/dL)	0.79 ± 0.86	0.360		
CRP (mg/L)	0.031 ± 0.03	0.39		
Sodium (mEq/L)	0.248 ± 0.30	0.410		
Cholesterol (mg/dL)	-0.020 ± 0.02	0.427		
DM (vs no DM)	-1.66 ± 2.14	0.44		

## Discussion

In the present study, we found higher IDWG% was associated with younger males, lower dry weight, and longer time on HD therapy. We speculate that younger patients drink more fluid because of increased physical and social activity, leading to higher IDWG%. Our results suggest that dietary advice, including fluid restriction, should be individualized based on age and body weight. In older patients, besides volume, multifactorial elements affect the blood pressure. Rahman et al. showed that high IDWG and younger age were independent predictors of higher BP and advancing age was associated with lower BP levels in this population [16]. We also found advancing age as a predictor of lower pre-dialysis systolic blood pressure but report that patients with the highest IDWG% did not have higher pre-dialysis systolic BP between these three groups (Table 5). BP was the same across all groups; this result likely is because the age of our population.

Our results showed that greater IDWG and IDWG% were not associated with better nutritional status. There was no significant difference in serum albumin, serum phosphorus (as a marker for protein intake in dialysis patients), and nPNA between the three groups. Some studies have reported that strict control of salt and fluids may affect nutritional status and lead to inadequate protein and calorie intake [17]. Ipema et al., in contrast with other studies [3, 5-6], did not find a strong association between IDWG and nutritional indicators, such as serum albumin and nPNA [18]. This study showed that there was a negative correlation between BMI and IDWG%. Sezer et al. has shown that patients with IDWG% > 3% had higher BMI than those who had an IDWG% < 3% [9].

Hecking and colleagues described that patients with high IDWG had low pre-dialysis sodium [19], which is similar to the results of the present study.

There was no significant association between IDWG and IDWG% with serum BUN, calcium-phosphorus (Ca x P) product, CRP, ferritin, anemia, Kt/V, and metabolic syndrome in studied patients. Regression analysis showed that increased hemoglobin can predict a 3% change in systolic blood pressure. 

## Conclusions

We did not find an association between IDWG% and increased systolic BP or nutritional status. Our results showed that age and body weight are important factors in IDWG and IDWG%. Being young is associated with higher IDWG and its percentage. Our findings highlight the importance of personalized advice on fluid and sodium restriction.
